# Tempol differently affects cellular redox changes and antioxidant enzymes in various lung-related cells

**DOI:** 10.1038/s41598-021-94340-z

**Published:** 2021-07-21

**Authors:** Woo Hyun Park

**Affiliations:** Department of Physiology, Medical School, Jeonbuk National University, 20 Geonji-ro, Deokjin-gu, Jeonju, Jeollabuk-do 54907 Republic of Korea

**Keywords:** Cancer, Cell biology

## Abstract

Tempol (4-hydroxy-2,2,6,6-tetramethylpiperidine-1-oxyl) is a potential redox agent in cells. The present study investigated changes in cellular reactive oxygen species (ROS) and glutathione (GSH) levels and in antioxidant enzymes, in Tempol-treated Calu-6 and A549 lung cancer cells, normal lung WI-38 VA-13 cells, and primary pulmonary fibroblasts. Results demonstrated that Tempol (0.5–4 mM) either increased or decreased general ROS levels in lung cancer and normal cells at 48 h and specifically increased O_2_^•−^ levels in these cells. In addition, Tempol differentially altered the expression and activity of antioxidant enzymes such as superoxide dismutase, catalase, and thioredoxin reductase1 (TrxR1) in A549, Calu-6, and WI-38 VA-13 cells. In particular, Tempol treatment increased TrxR1 protein levels in these cells. Tempol at 1 mM inhibited the growth of lung cancer and normal cells by about 50% at 48 h but also significantly induced cell death, as evidenced by annexin V-positive cells. Furthermore, down-regulation of TrxR1 by siRNA had some effect on ROS levels as well as cell growth inhibition and death in Tempol-treated or -untreated lung cells. In addition, some doses of Tempol significantly increased the numbers of GSH-depleted cells in both cancer cells and normal cells at 48 h. In conclusion, Tempol differentially increased or decreased levels of ROS and various antioxidant enzymes in lung cancer and normal cells, and induced growth inhibition and death in all lung cells along with an increase in O_2_^•−^ levels and GSH depletion.

## Introduction

The human lung is a structurally multidimensional organ and is susceptible to countless forms of injuries, which are risk factors for developing lung diseases like fibrosis and cancer^1^. As a rule, the physiological restoration process in a healthy lung is constantly active, and usually following injury will repair lung structure and restore function. On the other hand, the progression of lung repair can be pathological, leading to impaired structure and function. Pulmonary fibroblasts (PF) are fundamentally involved in repair and restoration following injuries^2^. During pathological recovery of the lung, sparse or redundant recruitment of fibroblasts can cause tissue dysfunction and eventually pulmonary disease^2^. Lung cancer is one of the most common lung diseases and one of the most important contributors to cancer-related mortality worldwide^3,4^. Lung cancer consists mainly of either small cell lung cancer (SCLC) or non-SCLC (NSCLC) types, which make up 10% to 13% and 85% to 90% of all lung cancer cases, respectively^3,4^. Existing drugs available are still inadequate, and this has prompted a demand for upgraded therapeutic approaches. Among the chemotherapy options tested are cytotoxic drugs that target the cell death signaling process (i.e. apoptosis or necrosis)^5–7^.

Reactive oxygen species (ROS) are very unstable oxygen molecules and include hydrogen peroxide (H_2_O_2_), hydroxyl radicals (^•^OH), and superoxide anions (O_2_^•−^) among others. These fundamental molecules are typically considered harmful to cells and tissues. However, ROS are contributory in regulating numerous cellular events such as gene expression, differentiation, and cell proliferation^8,9^. ROS, and in particular O_2_^•−^, are constantly generated during mitochondrial oxidative phosphorylation and unequivocally produced by specific oxidases including nicotinamide adenine dinucleotide phosphate (NADPH) oxidase and xanthine oxidase^10^. The main degradation pathway to reduce ROS levels employs superoxide dismutases [SODs; intracellular (SOD1), mitochondrial (SOD2), and extracellular (SOD3) isoforms], which metabolize O_2_^•−^ to H_2_O_2_^11^. H_2_O_2_ is then processed to O_2_ and/or H_2_O by catalase or glutathione (GSH) peroxidase^12^. GSH is an important antioxidant peptide which can protect cells from toxic insults^13^. In addition, thioredoxin (Trx) is a small antioxidant protein (~ 12 kDa) that has redox-active cysteine residues at its active site^14^. The oxidized form of Trx is reduced by NADPH-dependent Trx reductase (TrxR)^14^. While Trx1 and TrxR1 are usually localized in the cytoplasm, Trx2 and TrxR2 are found in mitochondria^14^. The Trx system is involved in cell survival, tumor development, and inflammatory diseases, particularly lung cancer^15–18^. Oxidative stress due to overproduction of ROS, a lack of antioxidants, or both, can lead to permanent modifications of proteins, lipids, and DNA, leading to cell death and tissue inflammation, consequently resulting in the chronic progression of many diseases including cancer^19,20^. More importantly, oxidative stress and chronic inflammation are associated with each other.

Tempol (4-hydroxy-2,2,6,6-tetramethylpiperidine-1-oxyl) is a synthetic cyclic nitroxide compound that has been commonly utilized as a contrast material in magnetic resonance spectroscopy^21,22^. It undergoes rapid reversible transfer between three forms: nitroxide, hydroxylamine and the oxoammonium cation form^21^. Therefore, Tempol is a potential redox agent that may function as a reductive or oxidative mediator depending on its concentration in the cell^21^. Treatment with Tempol removes a variety of ROS and diminishes oxidation, subsequently protecting cells and tissues from oxidative injuries^21,23–26^. Our previous reports have shown that Tempol prevents lung cancer cells and juxtaglomerular cells from undergoing cell death in response to cytotoxic insult^27,28^. Potential antioxidant mechanisms of Tempol have been proposed to explain these beneficial effects. One possibility is that it may act as a mimic of SOD and reduce the formation of ^•^OH either by scavenging O_2_^•−^ or by decreasing the intracellular concentration of Fe (II)^21,23,29^. However, increasing attention on Tempol in the clinical area has prompted more comprehensive studies on its potential toxicity. Abundant evidence suggests that Tempol interrupts ferritin synthesis and Fe metabolism, which could stimulate cell death^30^. The toxicity of Tempol is dose-dependent and preferentially targets cancer cells^31^. It has been reported that Tempol at millimole concentrations inhibits cell growth and induces apoptosis, depending on cell types and origins^31–33^. Moreover, high concentrations of, or extended exposure to Tempol increases ROS levels in juxtaglomerular cells^32^, breast cancer cells^34^, and ovarian cancer cells^33,35^. Thus, in order to clarify the different effects of Tempol as pro-oxidant or antioxidant in cells and tissues, further in depth studies need to be undertaken to elucidate its biological mechanisms.

In this study, alterations to cellular redox molecules such as ROS, GSH, and antioxidant enzymes in response to Tempol were examined in A549 and Calu-6 lung cancer cells, as well as in primary normal human PF (HPF) cells and an SV40-transformed normal HPF line (WI-38 VA-13 subclone 2RA). Moreover, it was examined whether down-regulation of Trx system, especially TrxR1 protein affects cell growth and death as well as ROS levels in Tempol-treated or -untreated lung cancer and normal cells.

## Materials and methods

### Cell culture

Human NSCLC A549 adenocarcinoma cells, SCLC Calu-6 cells, and normal lung WI-38 VA-13 subclone 2RA cells were obtained from the American Type Culture Collection (Manassas, VA, USA). Primary normal HPF cells were purchased from PromoCell GmbH (C-12360, Heidelberg, Germany) and used between passages five and ten. Lung cells were kept in a standard humidified incubator at 37 °C with 5% CO_2_ and cultured in RPMI-1640 medium supplemented with 10% fetal bovine serum (FBS; Sigma-Aldrich Co., St. Louis, MO, USA) and 1% penicillin–streptomycin (GIBCO BRL, Grand Island, NY, USA). Cells were grown in 100 mm plastic cell culture dishes (BD Falcon, Franklin Lakes, NJ, USA) and harvested with trypsin–EDTA (Gibco BRL).

### Reagents

Tempol was obtained from Sigma-Aldrich Co. and dissolved in methanol (Sigma-Aldrich Co.) at 1 M as a stock solution. 3-(4,5-dimethylthiazol-2-yl)-2,5-diphenyltetrazolium bromide (MTT) was also obtained from Sigma-Aldrich Co. and dissolved in distilled water. SOD and catalase proteins were acquired from Sigma-Aldrich Co. and dissolved in 50 mM potassium phosphate buffer at 4733 U/ml. Activity units of SOD and catalase were defined as the amount of enzyme needed to exhibit 50% dismutation of O_2_^•−^ and to exhibit 50% decomposition of H_2_O_2_ to H_2_O and/or O_2_ at 25 °C for 10 min, respectively, as previously described^36^. TrxR1 protein was also acquired from Sigma-Aldrich Co. All stock solutions were wrapped in foil and kept at 4 °C or -20 °C. Methanol (0.02%) was used as a vehicle control.

### Determination of intracellular ROS and O_2_^•−^ levels

Overall levels of intracellular ROS including H_2_O_2_, ^•^OH, and ONOO^•^ were measured using a fluorescent probe dye, 2',7'-dichlorodihydrofluorescein diacetate (H_2_DCFDA, Ex/Em = 495 nm/529 nm; Invitrogen Molecular Probes, Eugene, OR, USA), as previously described^37,38^. H_2_DCFDA is poorly sensitive to O_2_^•−^. However, dihydroethidium (DHE, Ex/Em = 518 nm/605 nm; Invitrogen Molecular Probes) is a fluorogenic probe that interacts selectively with O_2_^•−^^37^. In brief, 1 × 10^6^ cells in 60 mm culture dishes (BD Falcon) were treated with Tempol at indicated concentrations (0.5–4 mM) for 48 h. Then, the cells were washed in phosphate-buffered saline (PBS; GIBCO BRL) and incubated with 20 µM H_2_DCFDA or DHE at 37ºC for 30 min. The mean DCF and DHE fluorescence values were analyzed using a FAC Star flow cytometer (BD Sciences). The mean DCF and DHE levels are expressed as percentages compared to control cells.

### Western blot analysis

Expression levels of various antioxidant proteins were evaluated via western blotting. Briefly, 5 × 10^6^ cells in 100 mm culture dishes (BD Falcon) were incubated with the indicated concentrations of Tempol (0.5–4 mM) for 48 h. Cells were washed with PBS and four volumes of lysis buffer (Intron Biotechnology, Seongnam, Gyeonggi-do, Korea) were added. Total proteins (30 μg) were resolved in 12.5% SDS-PAGE gels and then transferred to Immobilon-P PVDF membranes (Millipore, Billerica, MA, USA) by electroblotting. Membranes were probed with anti-SOD1, anti-catalase, anti-Trx1, anti-TrxR1, and anti-β-actin (Santa Cruz Biotechnology, Santa Cruz, CA, USA). Membranes were incubated with horseradish peroxidase-conjugated secondary antibodies (Santa Cruz Biotechnology). Western blots were developed using an EZ-Western Lumi Pico ECL solution kit (DoGen, Seoul, Korea). All band intensities were analyzed using the ImageJ software program (FujiFilm, Tokyo, Japan).

### Measurement of cellular SOD activity

The activity of cellular SOD enzyme was measured using an SOD assay kit (Sigma-Aldrich Co.), as previously described^37^. In brief, 1 × 10^6^ cells in 60 mm culture dishes (BD Falcon) were incubated with 1 mM Tempol for 48 h. Supernatant samples that contained 30 μg protein were used for the measurement of SOD activity. These were added to wells of 96-well microtiter plates (Nunc, Roskilde, Denmark) with enzyme working solution containing a color reagent, and then the plates were incubated at 25 °C for 10 min to produce a water-soluble formazan dye. Formazan crystal formation was measured at 450 nm using a microplate reader (BioTekR Instruments Inc. Winooski, VT). SOD activity is expressed in arbitrary units.

### Measurement of cellular catalase activity

The activity of cellular catalase was measured using a catalase assay kit from Sigma-Aldrich Co., as previously described^37^. In brief, 1 × 10^6^ cells in 60 mm culture dishes (BD Falcon) were incubated with 1 mM Tempol for 48 h. Supernatant samples containing 30 μg protein were used for the determination of catalase activity. Samples were added to a microcentrifuge tube with assay buffer (50 mM KH_2_PO_4_/50 mM Na_2_HPO_4_, pH 7.0) and the reaction was commenced by the addition of 200 mM H_2_O_2_ solution at 25 °C for 5 min and ended by addition of stop solution. One hundred μl of the above reaction mixture was removed and added to another microcentrifuge tube with the color reagent (150 mM potassium phosphate buffer, pH7.0, containing 0.25 mM 4-aminoantipyrine, 2 mM 3,5-dichloro-2-hydroxybenzenesulfonic acid, and the peroxidase solution), and was incubated at 25 °C for 10 min. The dye was measured at 520 nm using a microplate reader (BioTekR Instruments Inc). Catalase activity is expressed in arbitrary units.

### Measurement of cellular TrxR1 activity

The activity of TrxR1 was assessed using a thioredoxin reductase assay kit according to the manufacturer's instructions (Sigma-Aldrich Co.). In brief, 1 × 10^6^ cells in 60 mm culture dishes (BD Falcon) were incubated with 1 mM Tempol for 48 h. Supernatant samples containing 30 μg protein were used for the determination of TrxR1 activity. These were added to wells of 96-well microtiter plates (Nunc) with 5,5'-dithiobis-(2-nitrobenzoic acid) from the kit at 25˚C for 1 h. The optical density of each well was measured at 412 nm using a microplate reader (BioTekR Instruments Inc). TrxR1 activity is expressed in arbitrary units.

### Transfection of cells with TrxR1 siRNAs

Gene silencing of TrxR1 was performed as previously described^39^. A control scrambled siRNA duplex [5’-CCUACGCCACCAAUUUCGU(dTdT)-3’] and TrxR1 siRNA duplex [5’-GUCGUCUAUGAGAAUGCUU(dTdT)-3’] were purchased from Bioneer Corp. (Daejeon, South Korea). In brief, 2 × 10^5^ cells added to six-well plates (Nunc) were incubated in RPMI-1640 supplemented with 10% FBS. The next day, cells (approximately 20–30% confluence) in each well were transfected with the control, or TrxR1 siRNA duplex [80 pmol in Opti-MEM (GIBCO BRL)] by means of LipofectAMINE 2000, according to the manufacturer’s instructions (Invitrogen, Branford, CT). One day later, cells were treated with or without 1 mM Tempol for 48 h. The transfected cells were used for Western blotting, MTT assays, annexin V-fluorescein isothiocyanate (FITC, Life Technologies, Carlsbad, CA, USA; Ex/Em = 488/519 nm) staining, and ROS measurements.

### Cell growth inhibition assay

The effects of Tempol on the growth of lung cells were determined using MTT assays. In brief, 1.5 × 10^4^ cells per well were seeded in 96-well microtiter plates (Nunc) for MTT assays. After incubation with Tempol at 1 mM for 48 h, 20 μL of MTT solution (2 mg/mL in PBS) was added to each well. Plates were then incubated at 37 °C for 3–4 h. Medium in each well was removed by pipetting. Then 100–200 μL of DMSO was added to each well to solubilize formazan crystals. Optical density was measured at 570 nm with a microplate reader (BioTekR Instruments Inc).

### Detection of apoptosis

Apoptosis was identified via annexin V-FITC (Life Technologies) as previously described^40^. Briefly, 3 × 10^5^–5 × 10^5^ cells in six-well plates (Nunc) were incubated with Tempol at 1 mM for 48 h. Cells were washed twice with cold PBS and then suspended in 200 μL of binding buffer (10 mM HEPES/NaOH pH 7.4, 140 mM NaCl, 2.5 mM CaCl_2_) at a density of 5 × 10^5^ cells/mL at 37 °C for 30 min. After adding Annexin V-FITC (2 μL), cells were analyzed with a FAC Star flow cytometer (BD Sciences).

### Detection of intracellular GSH levels

Cellular GSH levels were evaluated using fluorescent probe 5-chloromethylfluorescein diacetate (CMFDA, Ex/Em = 522 nm/595 nm; Invitrogen Molecular Probes), as previously described^37^. In brief, 1 × 10^6^ cells in 60 mm culture dishes (BD Falcon) were treated with Tempol at indicated concentrations (0.5–4 mM) for 48 h. Then, the cells were washed with PBS and incubated with 5 µM CMFDA at 37 ºC for 30 min. The mean CMF fluorescence intensity was determined using a FAC Star flow cytometer (BD Sciences). CMF negative (−) staining indicated the depletion of GSH content in cells.

### Statistical analysis

The results show the mean of more than two independent experiments (mean ± SD). The data were analyzed using Instat software (GraphPad Prism 5.0, San Diego, CA, USA). A Student’s *t*-test or one-way analysis of variance with post-hoc analysis using Tukey’s multiple comparison test was applied to determine statistical significance, which was defined as p-values of < 0.05.

## Results

### Effect of Tempol on intracellular ROS levels in lung cancer and normal cells

**Figure 1:**
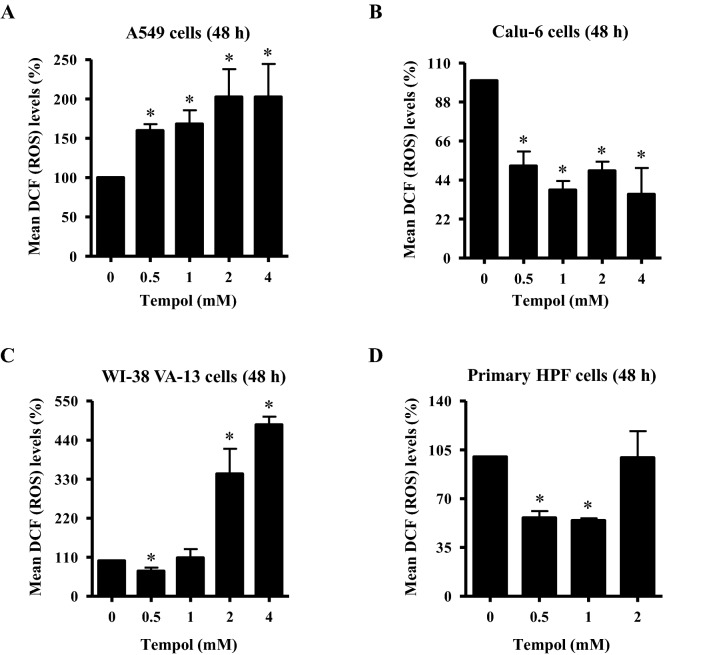
Effects of Tempol on intracellular ROS (DCF) levels in lung cancer and normal cells. Cells in the exponential growth phase were incubated with the indicated concentrations of Tempol for 48 h. Intracellular ROS (DCF) levels in lung cells were measured using a FAC Star flow cytometer. The graphs indicate mean ROS (DCF) levels (%) in A549 cells (**A**), Calu-6 cells (**B**), WI-38 VA-13 cells (**C**), and primary HPF cells (**D**). *p < 0.05 compared with untreated controls.

**Figure 2:**
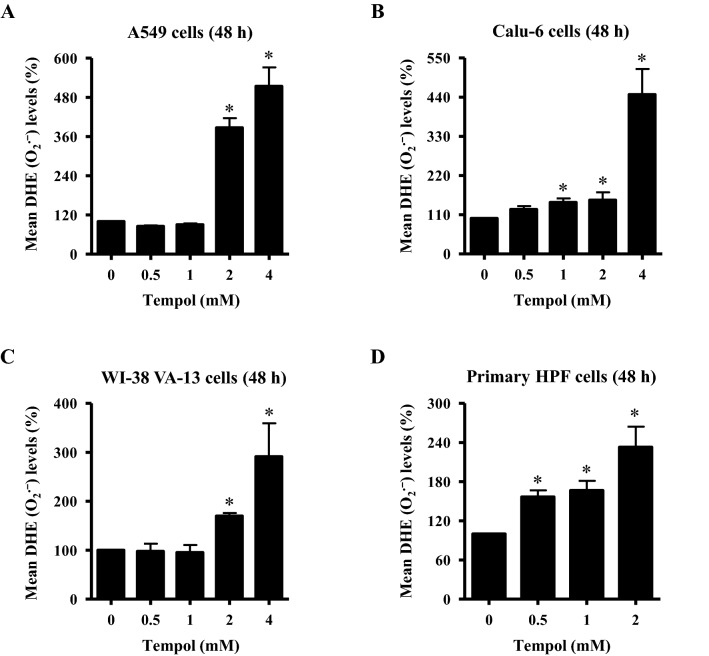
Effects of Tempol on intracellular DHE (O_2_^•−^) levels in lung cancer and normal cells. Cells in the exponential growth phase were incubated with the indicated concentrations of Tempol for 48 h. Intracellular DHE (O_2_^•−^) levels in lung cells were measured using a FAC Star flow cytometer. The graphs indicate mean DHE (O_2_^•−^) levels (%) in A549 cells (**A**), Calu-6 cells (**B**), WI-38 VA-13 cells (**C**), and primary HPF cells (**D**). *p < 0.05 compared with untreated controls.

To assess the intracellular levels of different kinds of ROS in Tempol-treated cells, H_2_DCFDA dye was used for general ROS levels and DHE dye was used for O_2_^•−^ levels. As shown in Fig. 1A, intracellular ROS (DCF) levels were significantly increased in A549 cells treated with 0.5–4 mM Tempol at 48 h. Doses of 1 and 4 mM Tempol increased the levels by about 170% and 200%, respectively, compared with the control group. In contrast, all tested doses of Tempol significantly decreased ROS (DCF) levels in Calu-6 cells (Fig. 1B). Doses of 1 and 4 mM Tempol decreased the levels by about 65% and 70%, respectively (Fig. 2B). For the normal lung cells, Tempol at 0.5 mM significantly decreased ROS (DCF) levels in WI-38 VA-13 cells at 48 h whereas 2 and 4 mM Tempol significantly increased ROS (DCF) levels in these cells, with 4 mM Tempol increasing the level by about 450% (Fig. 1C). While Tempol at 0.5 and 1 mM significantly decreased ROS (DCF) levels in primary HPF cells at 48 h by about 50%, 2 mM Tempol had no effect (Fig. 1D).

Intracellular O_2_^•−^ (DHE) levels were reduced in A549 cells treated with 0.5 and 1 mM Tempol at 48 h but they were significantly increased at 2 and 4 mM, with 4 mM Tempol increasing by about 500% (Fig. 2A). In Calu-6 cells, all Tempol doses increased O_2_^•−^ levels, with 1 and 4 mM Tempol increasing it by about 130% and 430%, respectively. In addition, Tempol at 2 and 4 mM significantly increased O_2_^•−^ levels in WI-38 VA-13 cells by about 160% and 290%, respectively (Fig. 2C). O_2_^•−^ levels were significantly increased in primary HPF cells treated with 0.5–2 mM Tempol at 48 h, with 2 mM Tempol promoting a 220% increase (Fig. 2D).

### Effects of Tempol on the expression and activity of various antioxidant enzymes in lung cancer and normal cell cells

**Figure 3:**
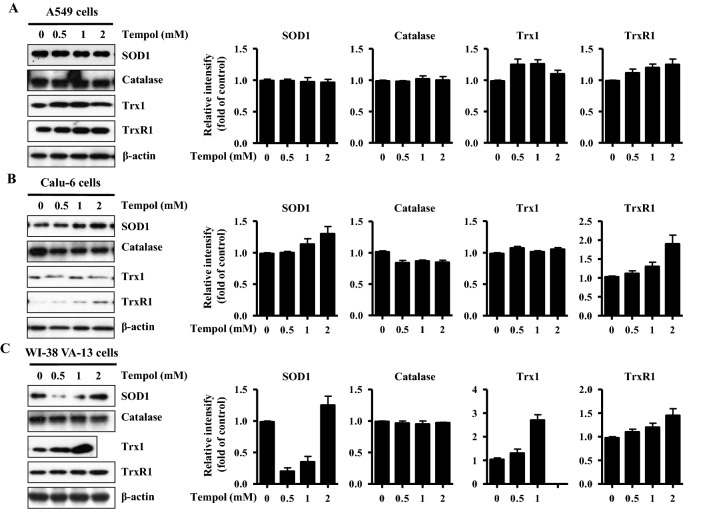
Effects of Tempol on the expression levels of SOD1, catalase, Trx1, and TrxR1 in lung cancer and normal cell cells. Exponentially growing cells were treated with indicated concentrations of Tempol for 48 h. Thirty μg of protein extracts from the tested lung cells were resolved by SDS-PAGE gel, transferred to PVDF membranes, and immunoblotted with the designated antibodies. Western blot analysis shows the levels of SOD1, catalase, Trx1, TrxR1, and β-actin in A549 cells (**A**), Calu-6 cells (**B**), and WI-38 VA-13 cells (**C**). The graphs represent the relative expression levels of SOD1, catalase, Trx1 and TrxR1 in A549 cells (**A**), Calu-6 cells (**B**), and WI-38 VA-13 cells (**C**). The expression of β-actin was used as an internal control.

Changing ROS levels result from alterations to antioxidant enzyme expression and activity. Thus, it was examined whether Tempol influenced the expression and activity of SOD, catalase, Trx1, and TrxR1 in lung cancer and normal cells at 48 h. As shown in Fig. 3A,B, and Supplementary Fig. 1A, Tempol at 0.5–2 mM did not affect SOD1 protein levels in A549 cells but increased the levels in Calu-6 cells. While 0.5 mM and 1 mM doses of Tempol diminished SOD1 protein levels in WI-38 VA-13 cells, 2 mM slightly increased the protein level (Fig. 3C and Supplementary Fig. 1A). For catalase, apparent differences in expression levels were not detected in Tempol-treated A549 and WI-38 VA-13 cells (Fig. 3A,C, and Supplementary Fig. 1B) whereas Tempol down-regulated catalase expression in Calu-6 cells (Fig. 3B and Supplementary Fig. 1B). In addition, Tempol increased Trx1 protein expression in A549 cells and WI-38 VA-13 cells (Fig. 3A,C, and Supplementary Fig. 1C) but did not affect Trx1 expression in Calu-6 cells (Fig. 3B and Supplementary Fig. 1C). Meanwhile increases in TrxR1 protein levels in response to Tempol were observed in all of these lung cells (Fig. 3 and Supplementary Fig. 1D).

**Figure 4:**
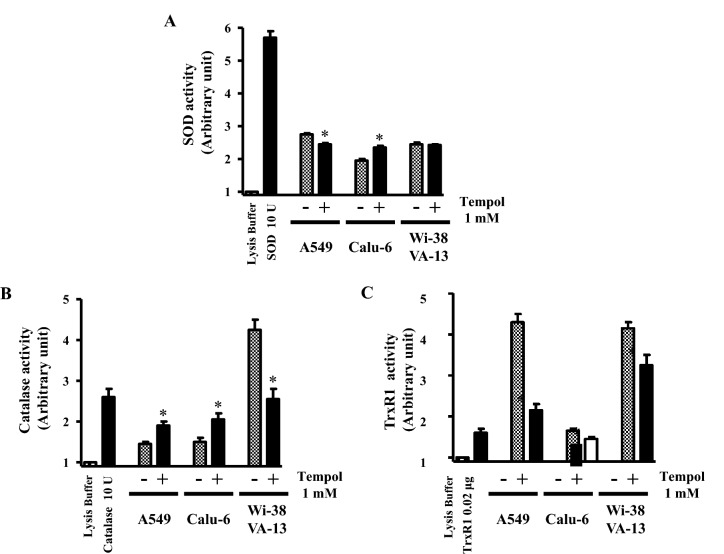
Effects of Tempol on SOD, catalase, and TrxR1 activity in lung cancer and normal cells. Exponentially growing cells were treated with 1 mM Tempol for 48 h. (**A**) The graph shows the activity of total SOD in arbitrary units in A549, Calu-6, and WI-38 VA-13 cells. Lysis buffer indicates a negative control in this assay. SOD 10 U indicates a positive control in this assay. (**B**) The graph shows the activity of catalase in arbitrary units in A549, Calu-6, and WI-38 VA-13 cells. Lysis buffer indicates a negative control in this assay. Catalase 10 U indicates a positive control in this assay. (**C**) The graph shows the activity of TrxR1 in A549, Calu-6, and WI-38 VA-13 cells. Lysis buffer indicates a negative control in this assay. TrxR1 0.02 μg indicates a positive control in this assay. *p < 0.05 compared with untreated controls.

Regarding the activity of these enzymes, it was found that the activity of SOD was down-regulated in A549 cells treated with 1 mM Tempol at 48 h while it was up-reregulated in 1 mM Tempol-treated Calu-6 cells (Fig. 4A). At 1 mM it did not affect the activity of SOD in WI-38 VA-13 cells (Fig. 4A). In addition, the activity of catalase was enhanced in Tempol-treated A549 and Calu-6 cells whereas it was significantly decreased in WI-38 VA-13 cells (Fig. 4B). Moreover, 1 mM Tempol significantly decreased the activity of TrxR1 at 48 h in all of these lung cells as compared to control cells (Fig. 4).

### Effects of TrxR1 silencing on cell growth, cell death, and ROS levels in Tempol-treated lung cancer and normal cells

**Figure 5:**
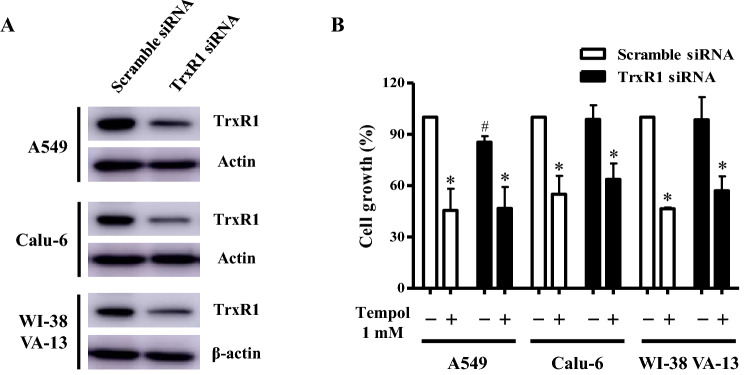
Effects of TrxR1 siRNA on cell growth in Tempol-treated lung cancer and normal cells. Cells (approximately 40–50% confluence) were transfected with control scramble siRNA or TrxR1 siRNA. One day later, cells were treated with 1 mM Tempol for 48 h. (**A**) Thirty μg of protein extracts from the tested lung cells were resolved by SDS-PAGE gel, transferred to PVDF membranes, and immunoblotted with TrxR1 and β-actin antibodies. Western blot analysis shows the levels of TrxR1 and β-actin in A549, Calu-6, and WI-38 VA-13 cells. (**B**) Cell growth was evaluated by MTT assays. Graph shows the growth of A549, Calu-6, and WI-38 VA-13 cells. *p < 0.05 compared with untreated controls. #p < 0.05 compared with scramble siRNA-transfected and Tempol-untreated A549 cells.

Because treatment with Tempol dose-dependently increased TrxR1 protein levels in A549, Calu-6, and Wi-38 VA-13 cells at 48 h (Fig. 3), it was assumed that changes in TrxR1 were closely related to cell growth and death as well as ROS levels. Hence whether gene silencing of TrxR1 using siRNA knock-down alters these cellular effects was examined. TrxR1 siRNA knock-down efficiently decreased protein levels more than 90% in all cell types at 48 h, as compared to scramble siRNA-transfected cells (Fig. 5A and Supplementary Fig. 2). Transfection of TrxR1 siRNA decreased the growth of A549 cells at 48 h by about 20% compared with control transfected A549 cells whereas cell growth was unaffected in Calu-6 and Wi-38 VA-13 cells (Fig. 5B). Treatment with 1 mM Tempol decreased the growth of scramble siRNA-transfected A549, Calu-6, and Wi-38 VA-13 cells by about 55%, 50%, and 55%, respectively (Fig. 5B). Tempol at 1 mM also reduced the growth of TrxR1 siRNA-transfected A549, Calu-6, and Wi-38 VA-13 cells by about 50%, 40%, and 50%, respectively (Fig. 5B).

**Figure 6:**
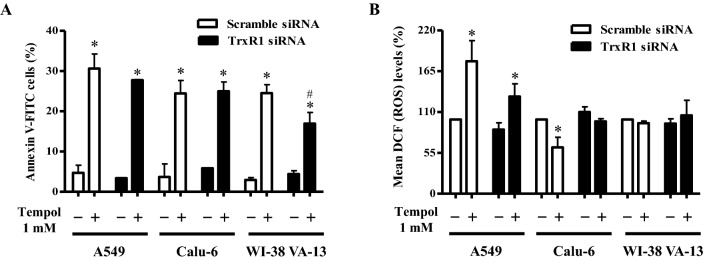
Effects of TrxR1 siRNA on cell death and ROS levels in Tempol-treated lung cancer and normal cells. Cells (approximately 40–50% confluence) were transfected with control scramble siRNA or TrxR1 siRNA. One day later, cells were treated with 1 mM Tempol for 48 h. (**A**) Annexin V-FITC positive cells were evaluated with a FAC Star flow cytometer. Graph shows proportions of annexin V-positive cells in A549, Calu-6, and WI-38 VA-13 cells. (**B**) Intracellular ROS (DCF) levels in the tested lung cells were measured using a FAC Star flow cytometer. Graph indicates mean ROS (DCF) levels (%) in A549, Calu-6, and WI-38 VA-13 cells. *p < 0.05 compared with untreated controls. #p < 0.05 compared with scramble siRNA-transfected and Tempol-treated WI-38 VA-13 cells.

In relation to cell death, transfection with TrxR1 siRNA did not significantly alter the proportion of annexin V-positive cells in untreated A549, Calu-6, and Wi-38 VA-13 cells at 48 h (Fig. 6A). However Tempol at 1 mM significantly increased the amounts of annexin V-positive cells in both scramble siRNA- and TrxR1 siRNA-transfected A549 cells by approximately 30% and 27%, respectively (Fig. 6A). In addition, Tempol increased the amounts of annexin V-positive cells in scramble siRNA- and TrxR1 siRNA-transfected Calu-6 cells by approximately 24% (Fig. 6A). Interestingly, it augmented the proportion of annexin V-positive cells in scramble siRNA- and TrxR1 siRNA-transfected Wi-38 VA-13 cells by approximately 22% and 17%, respectively, implying that knock-down of TrxR1 attenuates cell death in Tempol-treated Wi-38 VA-13 cells (Fig. 6A). With respect to ROS levels, TrxR1 silencing slightly decreased ROS levels in A549 cells at 48 h and slightly increased ROS levels in Calu-6 cells (Fig. 6B). Tempol at 1 mM significantly increased ROS levels in scramble siRNA- and TrxR1 siRNA-transfected A549 cells at 48 h by approximately 175% and 130%, respectively (Fig. 6B). In contrast, 1 mM Tempol decreased ROS levels in scramble and TrxR1 siRNA-transfected Calu-6 cells by approximately 40% and 15%, respectively (Fig. 6B), while in Wi-38 VA-13 cells, it did not significantly change ROS levels (Fig. 6B).

### Effect of Tempol on GSH levels in lung cancer and normal cells

**Figure 7:**
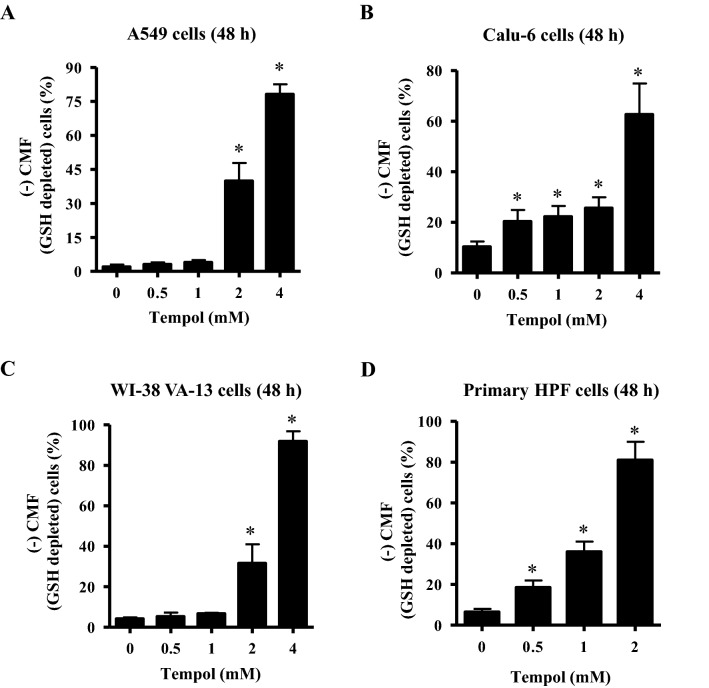
Effects of Tempol on intracellular GSH depletion in lung cancer and normal cells. Cells in the exponential growth phase were incubated with the indicated concentrations of Tempol for 48 h. Intracellular CMF (GSH) levels in various lung cells were measured using a FAC Star flow cytometer. The graphs indicate the percentages of (–) CMF (GSH-depleted) in A549 cells (**A**), Calu-6 cells (**B**), WI-38 VA-13 cells (**C**), and primary HPF cells (**D**). *p < 0.05 compared with untreated controls.

Changes in GSH levels were assessed in lung-related cells using CMF fluorescent dye. As shown in Fig. 7A, Tempol at 0.5 and 1 mM did not augment the number of GSH-depleted cells in Calu-6 cells at 48 h, whereas 2 and 4 mM concentrations significantly increased the number of GSH-depleted cells by about 44% and 74%, respectively. Tempol at 0.5–4 mM significantly increased the number of GSH-depleted cells in A549 cells at 48 h, with 1 and 4 mM Tempol increasing numbers by about 20% and 60%, respectively (Fig. 7B). In WI-38 VA-13 cells, 0.5 and 1 mM Tempol did not affect the number of GSH-depleted cells but 2 and 4 mM concentrations significantly augmented the number of GSH-depleted cells by about 28% and 90%, respectively (Fig. 7C). In addition, Tempol significantly induced GSH depletion in primary HPF cells at 48 h, with 0.5, 1, and 3 mM Tempol increasing the depletion by about 19%, 38%, and 80%, respectively (Fig. 7D).

## Discussion

Tempol has been shown to protect cells and tissues from oxidative damage^21,23–26^. In contrast, it has also been reported that Tempol enhances inflammation and oxidative damage in numerous cell and tissue models^21,23^. In addition, millimole concentrations of Tempol or extended exposure to it increases ROS levels in juxtaglomerular cells^32^, breast cancer cells^34^, and ovarian cancer cells^33,35^. The current study has focused on investigating changes in cellular redox changes and antioxidant enzymes in Tempol-treated lung cancer and normal cells.

Results from the present study showed that ROS (DCF) levels were significantly increased in A549 cells treated with 0.5–4 mM Tempol and in WI-38 VA-13 cells treated with 2 and 4 mM Tempol. However, ROS (DCF) were significantly reduced in Calu-6 cells treated with 0.5–4 mM Tempol and in WI-38 VA-13 cells treated with 0.5 mM Tempol. In addition, Tempol at 0.5 and 1 mM significantly decreased ROS (DCF) in primary HPF cells. These results imply that Tempol can raise or lower ROS (DCF) levels depending on the cell type without significant differences between lung cancer and normal lung cells. Tempol at 1 mM significantly increased the activity of catalase in Calu-6 cells, possibly resulting in a decrease in intracellular H_2_O_2_ level due to the high conversion from H_2_O_2_ to O_2_ and H_2_O. However, the increased activity of catalase in Tempol-treated A549 cells did not reduce ROS (DCF) levels and the decreased activity of catalase in Tempol-treated WI-38 VA-13 cells did not increase ROS (DCF). In addition, Tempol did not apparently alter the expression of catalase in A549 and WI-38 VA-13 cells and decreased catalase in Calu-6 cells. Therefore, the expression levels of catalase in lung cancer and normal cells did not correlate with its enzymatic activity.

Intracellular O_2_^•−^ (DHE) levels were increased in A549, Calu-6, WI-38 VA-13, and primary HPF cells treated with 2 or 4 mM Tempol. Tempol also increased O_2_^•−^ levels in Calu-6 and primary HPF cells at 0.5 and 1 mM but these doses did not augment O_2_^•−^ levels in A549 and WI-38 VA-13 cells. Relatively high concentrations of Tempol appeared to increase O_2_^•−^ in each of the cell lines. Tempol is a mimic of SOD which metabolizes O_2_^•−^ to H_2_O_2_^21,29^. Similarly, 1 mM Tempol up-regulated the activity of SOD in Calu-6 cells at 48 h. However, Tempol did not affect the activity of SOD in WI-38 VA-13 cells. Tempol at 1 mM even reduced the activity of SOD in A549 cells. Treatment with Tempol increased SOD1 protein levels in Calu-6 cells but not in A549 cells. Contrastingly, at 1 mM it decreased the levels in WI-38 VA-13 cells. Therefore, the expression levels of SOD1 in A549 and WI-38 VA-13 cells were not related to its enzymatic activity. It was speculated that the increased amount of intracellular O_2_^•−^ in cells in response to Tempol may have been originated from mitochondria. Numerous reports have demonstrated that Tempol mediates its toxicity via the disruption of mitochondrial function^32,33,41^, suggesting that Tempol’s effects on mitochondria may result in excessive production of O_2_^•−^, causing oxidative stress and consequently apoptosis. In fact, 2 and 4 mM of Tempol strongly increased mitochondrial O_2_^•−^ levels in WI-38 VA-13 cells at 48 h, by approximately sixfold and 22-fold, respectively (Supplementary Fig. 3). In lung cancer and normal cells, death from Tempol was more correlated with changes in O_2_^•−^ levels than ROS (DCF) levels. The apparent diverse effects of Tempol in different conditions and in different cells could be explained by assuming that each cell has a different basal activity or metabolism of mitochondria and a variety of antioxidant enzymes present in the cytoplasm or other organelles.

The Trx system consists of Trx, TrxR, and NADPH and is a fundamental enzymatic complex that preserves cellular redox homeostasis^14^. Trx1 and/or TrxR1 are overexpressed in various pulmonary diseases including cancer^16,42–44^. Hence, it is plausible that the Trx system is a promising target for treatment of lung cancer. Tempol significantly decreased the activity of TrxR1 in A549, Calu-6, and Wi-38 VA-13 cells. However, TrxR1 protein levels were increased in all these lung cells. Thus, the increased activity of TrxR1 in Tempol-treated lung cells is more likely to be attributed to other factors such as Trx and Trx-interacting proteins rather than simple expression levels. In addition, Tempol treatment increased Trx1 protein expression in A549 cells and WI-38 VA-13 cells but not in Calu-6 cells. Further research is needed on the mechanism underlying alterations in the Tempol-mediated Trx system.

Treatment of Tempol at 1–5 mM inhibits cell growth and induces apoptosis in various cancer cells^32–35,41,45^. Likewise, 1 mM Tempol reduced the growth of scramble siRNA-transfected A549 and Calu-6 lung cancer cells, and Wi-38 VA-13 normal cells by about 55%, 50%, and 55% at 48 h, respectively. Moreover, Tempol at 1 mM also significantly increased the amounts of annexin V-positive cells in scramble siRNA-transfected A549, Calu-6, and Wi-38 VA-13 cells by about 30%, 24%, and 22% at 48 h, respectively. Therefore, Tempol is likely to induce lung cell death via apoptosis and/or necrosis. There was no significant difference in the susceptibility to Tempol among these lung cancer and normal cells. TrxR1 silencing itself did not influence the growth of Calu-6 and Wi-38 VA-13 cells compared with scramble siRNA-transfected control cells but it did inhibit the growth of A549 control cells by about 20%. Transfection of TrxR1 siRNA did not significantly alter the growth inhibition in both Tempol-treated lung cancer and normal cells. Thus, reduced TrxR1 protein level was involved in growth inhibition only in A549 cells among these lung cells and did not affect growth inhibition in these lung cells treated with Tempol. In addition, TrxR1 siRNA alone did not increase the amounts of annexin V-positive cells in A549, Calu-6, and Wi-38 VA-13 control cells. Furthermore, knock-down of TrxR1 slightly decreased cell death in Tempol-treated A549 cells and significantly attenuated cell death in Tempol-treated Wi-38 VA-13 cells. These results suggest that TrxR1 silencing does not have a direct effect on cell survival in Tempol-untreated control lung cells, but has a differential effect on cell survival in various lung cells treated with Tempol.

A549 cells transfected with TrxR1 siRNA showed slightly decreased ROS (DCF) levels at 48 h while transfected Calu-6 cells showed slight increases. TrxR1 siRNA reduced the increased levels of ROS (DCF) in Tempol-treated A549 cells whereas silencing partially recovered the decreased levels of ROS (DCF) in Tempol-treated Calu-6 cells. In Wi-38 VA-13 cells, TrxR1 siRNA did not alter ROS (DCF) levels regardless of 1 mM Tempol treatment. It has been reported that Trx or TrxR1 could regulate the basal cellular metabolism of glycolysis, tricarboxylic acid cycle, and mitochondria to produce adenosine triphosphate (ATP) or NAD(P)H^46^. In addition, there are potentially harmful interactions between Tempol and enzyme complexes, which are inolved in glucose transport, glutamine metabolism and mitochondiral ATP production^21,23,33^. Therefore, further studies are needed on the difference in cellular ROS levels in lung cancer and normal cells following downregulation of TrxR1 and treatment with Tempol.

Depletion of GSH promotes cell death^47–49^ and this has been reported to occur in Tempol-treated leukemia and cervical cancer cells^41,50^. Similarly, the present study demonstrated an increased proportion of GSH-depleted cells in A549, Calu-6, WI-38 VA-13, and primary HPF cells treated with 2 or 4 mM Tempol. In addition, lower doses of Tempol (0.5 and 1 mM) significantly induced GSH-depleted cells in Calu-6 and primary HPF cells. These results support the notion that the induction of cell death was inversely proportional to GSH content^47–49^. However, 1 mM Tempol, which showed cell growth inhibition and cell death in A549 and WI-38 VA-13 cells, did not significantly augment the number of GSH-depleted cells in these cells. As there was little difference in susceptibility to cell death to Tempol between lung cancer and normal cells, the effect of GSH depletion by Tempol was not significantly different between lung cancer and normal cells. According to the present study, GSH content is a crucial factor or indicator of cell death induced by Tempol, but its content alone is not sufficient to accurately predict cell death.

In summary, the present results have shown that Tempol at 0.5–4 mM could either increase or decrease ROS (DCF) levels in lung cancer and normal cells and this drug specifically increased O_2_^•−^ levels and GSH depletion. Tempol at 1 mM inhibited the growth of lung cancer and normal cells and also induced their cell death. There was no significant difference in Tempol sensitivity between lung cancer and normal cells. In addition, Tempol had other effects on the expression and activity of antioxidant enzymes, especially TrxR1 in lung cancer and normal cells. Furthermore, down-regulation of TrxR1 partially affected cell growth and death, as well as ROS (DCF) levels in both Tempol-untreated or -treated cells, depending on the cell type. The current results provide valuable information for understanding the cellular effects of Tempol on lung cancer and normal cells with regard to cell growth inhibition and cell death, as well as redox states (ROS and GSH levels) and various antioxidant pathways.

### Ethics declarations

The material in this paper has not been published or is not under active consideration by another journal. The research was conducted in accordance with the declaration of Helsinki.

## Supplementary Information

Supplementary Information 1.

Supplementary Information 2.

## Data Availability

Data collected during the present study are available from the corresponding author upon reasonable request.

## Abbreviations

NSCLC: Non-small cell lung cancer
SCLC: Small cell lung cancer
HPF: Human pulmonary fibroblast
ROS: Reactive oxygen species
GSH: Glutathione
Trx: Thioredoxin
TrxR: Trx reductase
SOD: Superoxide dismutase
NADPH: Nicotinamide adenine dinucleotide phosphate
H_2_DCFDA: 2',7'-Dichlorodihydrofluorescein diacetate
DHE: Dihydroethidium
CMFDA: 5-Chloromethylfluorescein diacetate
MTT: 3-(4,5-Dimethylthiazol-2-yl)-2,5-diphenyltetrazolium bromide
FITC: Fluorescein isothiocyanate
