# Supplementary material for: Tempol differently affects cellular redox changes and antioxidant enzymes in various lung-related cells
Source: Sci Rep. 2021 Jul 21;11:14869. doi: 10.1038/s41598-021-94340-z (PMC8295274; doi:10.1038/s41598-021-94340-z)
Supplement: Supplementary file 1 — Supplementary Information 1. [file 41598_2021_94340_MOESM1_ESM.ppt]

## Slide 1
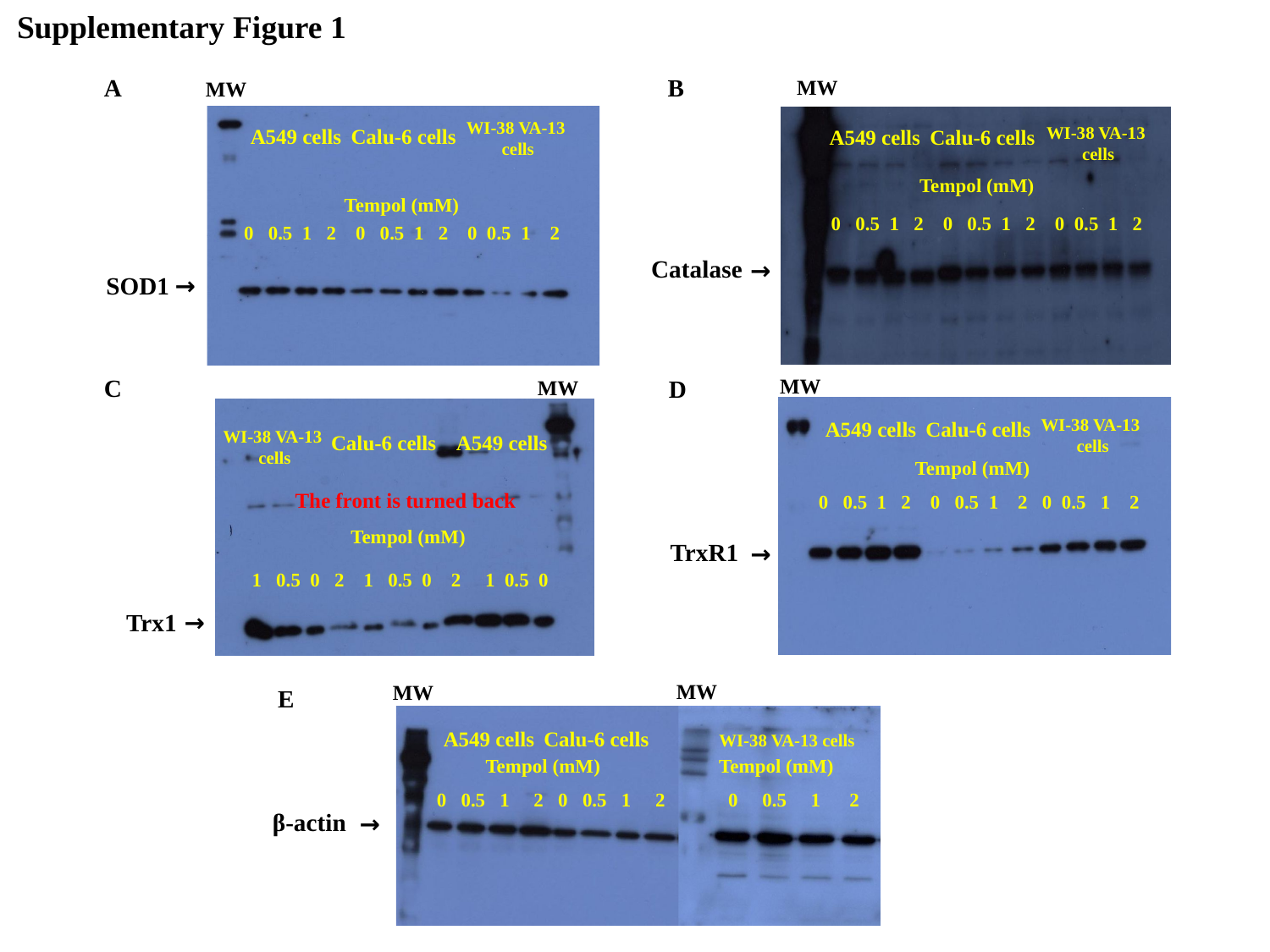

Supplementary Figure 1
A
B
MW
MW
WI-38 VA-13
cells
WI-38 VA-13
cells
A549 cells
Calu-6 cells
A549 cells
Calu-6 cells
Tempol (mM)
Tempol (mM)
0 0.5 1 2 0 0.5 1 2 0 0.5 1 2
0 0.5 1 2 0 0.5 1 2 0 0.5 1 2
0 0.5 1 2
Catalase
→
SOD1
→
C
MW
D
MW
WI-38 VA-13
cells
A549 cells
Calu-6 cells
WI-38 VA-13
cells
Calu-6 cells
A549 cells
Tempol (mM)
The front is turned back
0 0.5 1 2 0 0.5 1 2 0 0.5 1 2
Tempol (mM)
TrxR1
→
1 0.5 0 2 1 0.5 0 2 1 0.5 0
Trx1
→
MW
MW
E
A549 cells
Calu-6 cells
WI-38 VA-13 cells
Tempol (mM)
Tempol (mM)
0 0.5 1 2 0 0.5 1 2 0 0.5 1 2
β-actin
→

## Slide 2
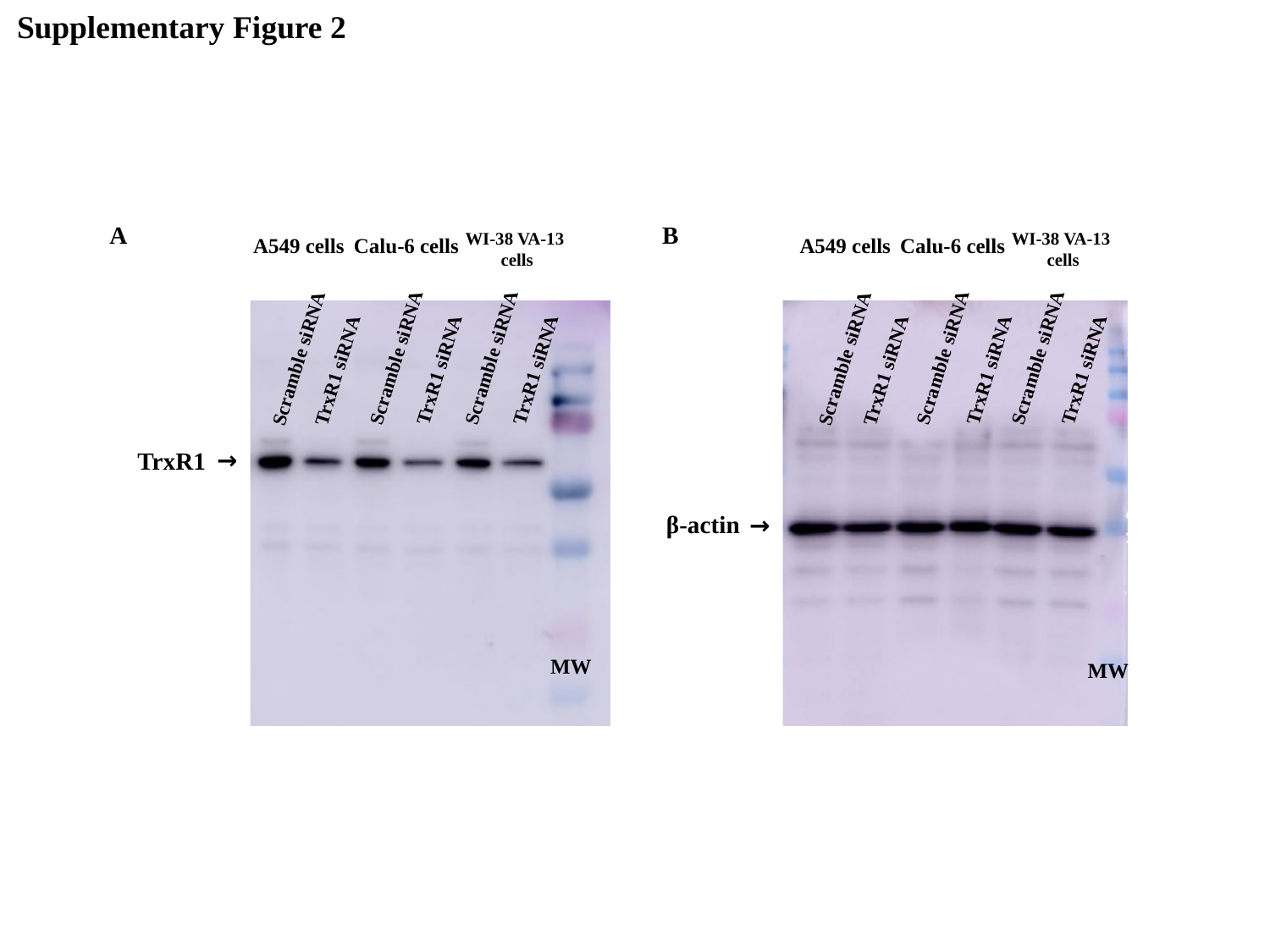

Supplementary Figure 2
A
B
WI-38 VA-13
cells
WI-38 VA-13
cells
A549 cells
Calu-6 cells
A549 cells
Calu-6 cells
Scramble siRNA
TrxR1 siRNA
Scramble siRNA
TrxR1 siRNA
TrxR1 siRNA
TrxR1 siRNA
Scramble siRNA
Scramble siRNA
TrxR1 siRNA
TrxR1 siRNA
Scramble siRNA
Scramble siRNA
→
TrxR1
β-actin
→
MW
MW

## Slide 3
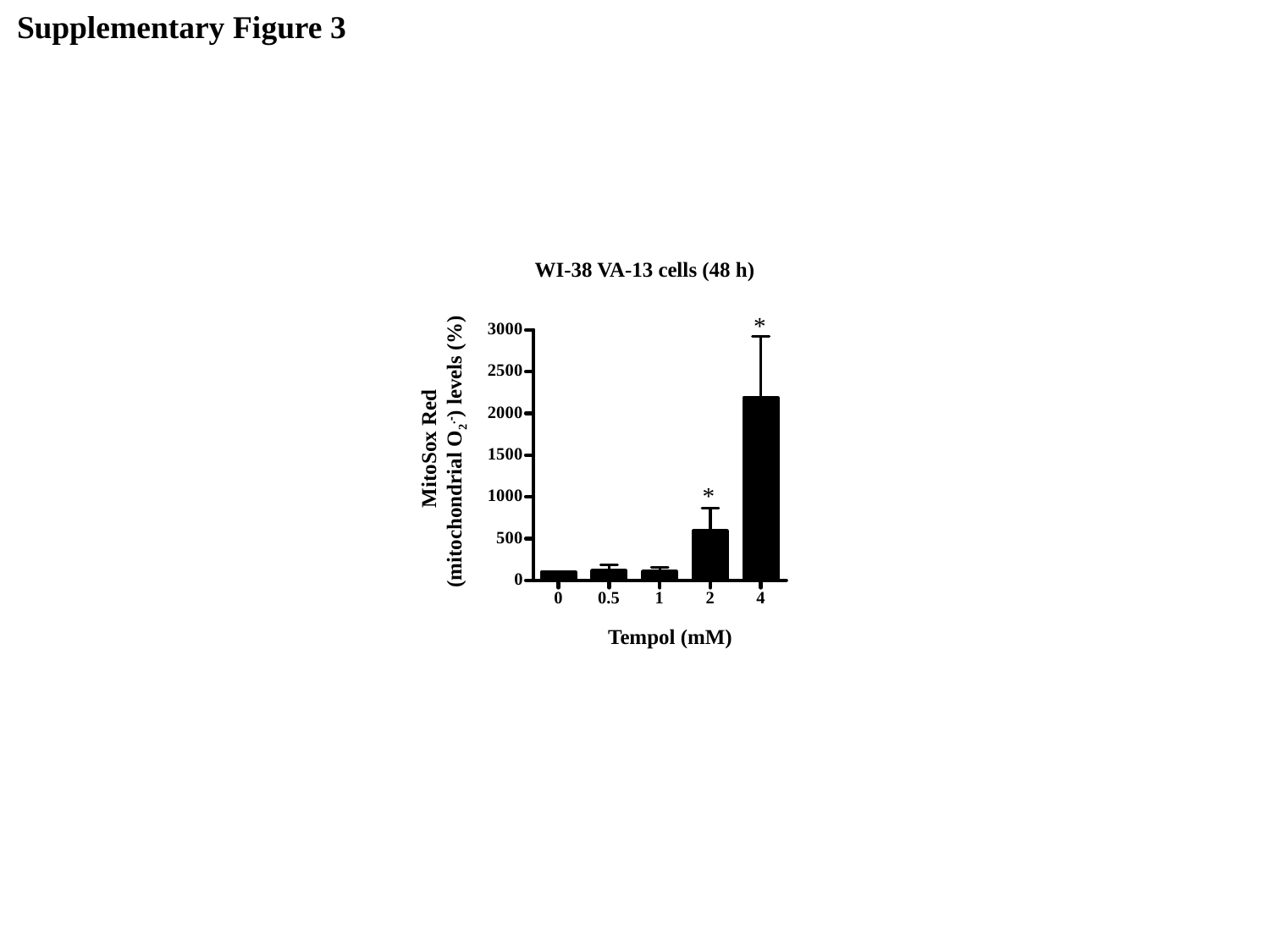

Supplementary Figure 3
WI-38 VA-13 cells (48 h)
*
MitoSox Red
(mitochondrial O2.-) levels (%)
*
Tempol (mM)
