# Supplementary material for: Tempol differently affects cellular redox changes and antioxidant enzymes in various lung-related cells
Source: Sci Rep. 2021 Jul 21;11:14869. doi: 10.1038/s41598-021-94340-z (PMC8295274; doi:10.1038/s41598-021-94340-z)
Supplement: Supplementary file 2 — Supplementary Information 2. [file 41598_2021_94340_MOESM2_ESM.docx]

**Legends to supplementary figures**

**Supplementary Figure 1.** Effects of Tempol on the expression levels of SOD1, catalase, Trx1, and TrxR1 in lung cancer and normal cell cells. Exponentially growing cells were treated with indicated concentrations of Tempol for 48 h. Thirty μg of protein extracts from the tested lung cells were resolved by SDS-PAGE gel, transferred to PVDF membranes, and immunoblotted with the designated antibodies. Western blot analysis shows the levels of SOD1 (A), catalase (B), Trx1 (C), TrxR1 (D), and β-actin (E) in A549 cells, Calu-6 cells, and WI-38 VA-13 cells. MW stands for molecular weight.

**Supplementary Figure 2.** Effects of TrxR1 siRNA on the expression levels of TrxR1 and β-actin in lung cancer and normal cell cells. Cells (approximately 40 ~ 50% confluence) were transfected with control scramble siRNA or TrxR1 siRNA. Three day later, 30 μg of protein extracts from the tested lung cells were resolved by SDS-PAGE gel, transferred to PVDF membranes, and immunoblotted with TrxR1 and β-actin antibodies. Western blot analysis shows the levels of TrxR1 (A) and β-actin (B) in A549, Calu-6, and WI-38 VA-13 cells. MW stands for molecular weight.

**Supplementary Figure 3.** Effects of Tempol on intracellular MitoSox Red (mitochondrial O_2_^•−^ ) levels in WI-38 VA-13 cells. Cells in the exponential growth phase were incubated with the indicated concentrations of Tempol for 48 h. Intracellular MitoSox Red (mitochondrial O_2_^•−^ ) levels in lung cells were measured using a FAC Star flow cytometer. The graphs indicate mean MitoSox Red (mitochondrial O_2_^•−^ ) levels (%) in WI-38 VA-13 cells. *p < 0.05 compared with untreated control.
